# Species diversity of pathogenic wood-rotting fungi (Agaricomycetes, Basidiomycota) in China

**DOI:** 10.1080/21501203.2023.2238779

**Published:** 2023-08-08

**Authors:** Yuan Yuan, Lu-Sen Bian, Ying-Da Wu, Jia-Jia Chen, Fang Wu, Hong-Gao Liu, Guang-Yu Zeng, Yu-Cheng Dai

**Affiliations:** a Institute of Microbiology, School of Ecology and Nature Conservation, Beijing Forestry University, Beijing, China; b Experimental Centre of Forestry in North China, Warm Temperate Zone Forestry Jiulong Mountain National Permanent Scientific Research Base, Chinese Academy of Forestry, Beijing, China; c Key Laboratory of Forest and Grassland Fire Risk Prevention, Ministry of Emergency Management, China Fire and Rescue Institute, Beijing, China; d College of Landscape Architecture, Jiangsu Vocational College of Agriculture and Forestry, Zhenjiang, China; e Yunnan Key Laboratory of Gastrodia and Fungi Symbiotic Biology, Zhaotong University, Zhaotong, China; f Guangxi Forestry Science Research Institute, Nanning, China

**Keywords:** Basidiomycota, forest diseases, tree pathogens, wood-rotting fungi

## Abstract

Wood-rotting basidiomycetes have been investigated in the Chinese forest ecosystem for the past 30 years. Two hundred and five pathogenic wood-decayers belonging to 9 orders, 30 families, and 74 genera have been found in Chinese native forests, plantations, and gardens. Seventy-two species (accounting for 35% of the total pathogenic species) are reported as pathogenic fungi in China for the first time. Among these pathogens, 184 species are polypores, nine are corticioid fungi, eight are agarics and five are hydnoid basidiomycetes. One hundred and seventy-seven species (accounting for 86%) cause white rot, while 28 species (accounting for 14%) result in brown rot; 157 species grow on angiosperm trees (accounting for 76.5%) and 44 species occur on gymnosperm trees (accounting for 21.5%), only four species inhabit both angiosperms and gymnosperms (accounting for 2%); 95 species are distributed in boreal to temperate forests and 110 in subtropical to tropical forests. In addition, 17 species, including *Fomitopsis pinicola*, *Heterobasidion parviporum*, and *Phellinidium weirii* etc. which were previously treated as pathogenic species in China, do not occur in China according to recent studies. In this paper, the host(s), type of forest, rot type, and distribution of each pathogenic species in China are given.

## 1. Introduction

Wood-rotting basidiomycetes in Chinese forests have been extensively studied during the past 30 years, and almost all types of forest vegetation in all provinces have been surveyed. Nearly 1,600 species of wood-rotting basidiomycetes have been found in China (Dai 2011, 2012; Cui et al. 2019; Sun et al. 2019, 2022; Li and Wei 2021; Dai et al. 2021, 2021, 2022; Liu et al. 2021, 2023; Ma et al. 2022; Wu et al. 2022). The majority of wood-rotting basidiomycetes are saprophytic and utilise dead wood as a substrate for growth and reproduction. However, some wood-rotting species attack living trees by either invading and killing the living sapwood or living in the heartwood of living hosts. Although the latter are restricted to the non-living part of the tree, they cause major timber losses for sawmills, in addition, more losses can occur as the infected trees can be further damaged due to windthrow (Dai et al. 2007). Both types of wood-rotting basidiomycetes found in China, i.e. those that cause white rot and those that cause brown rot, are included in the present study. We include species that prefer dead or living trees but also species that usually grow on dead wood but occasionally attack living trees when the tree is in a weakened state due to age, fire damage, insect damage, other fungal attacks etc.

A special genus of wood-rotting basidiomycetes is *Phylloporia* Murrill, the species of which exhibit high levels of host specificity for living trees or bushes. The particular feature of *Phylloporia* is that some species in this genus do not decay host wood immediately, but rather coexist with the host for a period of time. Other *Phylloporia* species slowly decay root, heartwood, or sapwood of hosts, causing damage to their hosts. Therefore, we treat *Phylloporia* species in the latter case as forest pathogens.

The species dealing with this paper is mostly the so-called aphyllophoid basidiomycetes, but a few important agarics, e.g. *Armillaria*, are included.

Previously, 102 (Dai et al. 2007) and 152 (Dai 2012) pathogenic wood-rotting species were reported by the senior author. However, phylogenetic analyses on wood-rotting pathogens indicated that many common species are species complexes. For instance, *Laetiporus sulphureus* (Bull.) Murrill was considered a common species in the Northern Hemisphere (Núñez and Ryvarden 2001; Ryvarden and Melo 2014; Zhou et al. 2016), but recent studies showed that four additional new species could be derived from *Laetiporus sulphureus* (Song et al. 2014; Liu et al. 2023). Another example, *Fomitopsis pinicola* (Sw.) P. Karst. was recorded as a common species in China (Dai 2012), but recent phylogenetic analyses found that five species were derived from *Fomitopsis pinicola* sensu lato, and Fomitopsis pinicola sensu stricto is distributed in Europe only. In addition, recent studies reported new pathogenic wood-rotting basidiomycetes from China (Wang and Dai 2022; Wu et al. 2022; Yuan et al. 2022; Zhao et al. 2022). Furthermore, the systematics of wood-rotting basidiomycetes has changed with the advancement in molecular techniques, and many new genera have been established with numerous combinations proposed (Cui et al. 2019; Sun et al. 2019, 2022; Wu et al. 2022; Liu et al. 2023). More than 75% of the scientific names of the Chinese pathogenic wood-rotting species in the previous lists have changed according to the modern taxonomy, and 72 new pathogenic wood-rotting basidiomycetes have been recorded in China since the previous publication on the diversity of wood-inhabiting fungi (Dai 2012). This was the impetus for this current update on the diversity of pathogenic wood-rotting basidiomycetes in China.

## 2. Materials and methods

The data are mostly based on our investigations, sampling, and observations. Extensive field inventories were made of almost all the Chinese forests in all the provinces during the past 30 years; about 30,000 samples were collected, and nearly 2,000 culture isolates were made. Species identification is based on morphology and molecular work, and the voucher specimens are deposited in the fungoria of Beijing Forestry University (BJFU) and the Institute of Applied Ecology, Chinese Academy of Sciences (IFP). A minor number of species mentioned as forest pathogens in other publications are included in the present (Zhou 1981; Anonymous 1983; Jing et al. 1988; Ren 1993; Yuan 1997).

## 3. Results

In the following list, the pathogenic wood-rotting basidiomycetes are arranged alphabetically according to genus and the species within the genera. The scientific names and their authors are mostly according to Index Fungorum (https://www.indexfungorum.org/names/names.asp) and MycoBank (https://www.mycobank.org/). However, some updated scientific names are according to our recent studies (Cui et al. 2019; Wu et al. 2022; Yuan et al. 2022).

**Figure 1.**
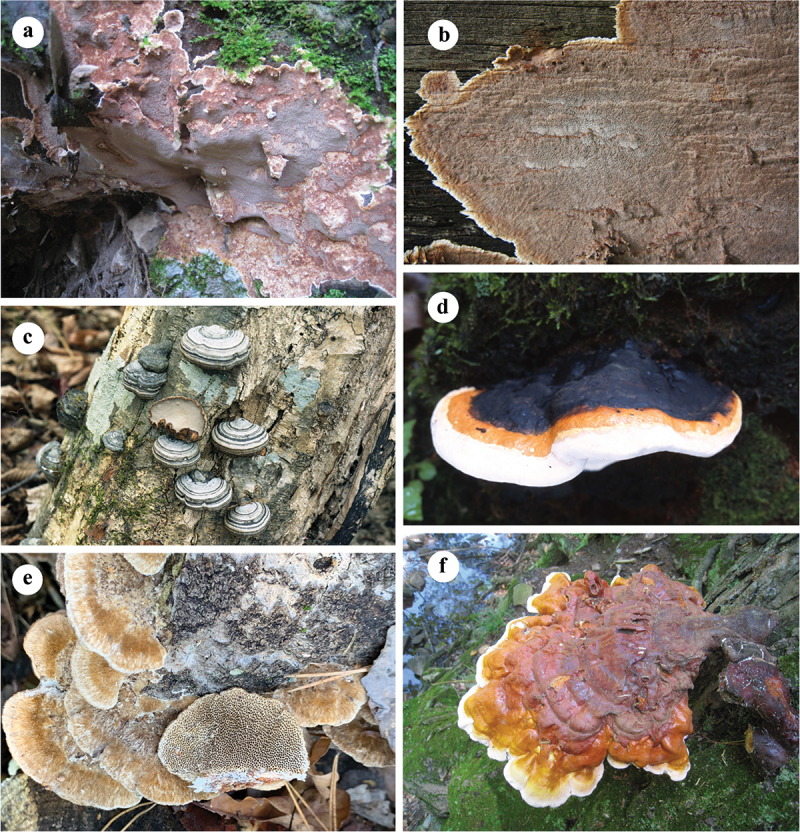
Basidiomata of some typical forest pathogens. (a) *Coniferiporia qilianensis*. (b) *Coniferiporia sulphurascens*. (c) *Fomes fomentarius*. (d) *Fomitopsis subpinicola*. (e) *Funalia trogii*. (f) *Ganoderma tsugae*.

**Figure 2.**
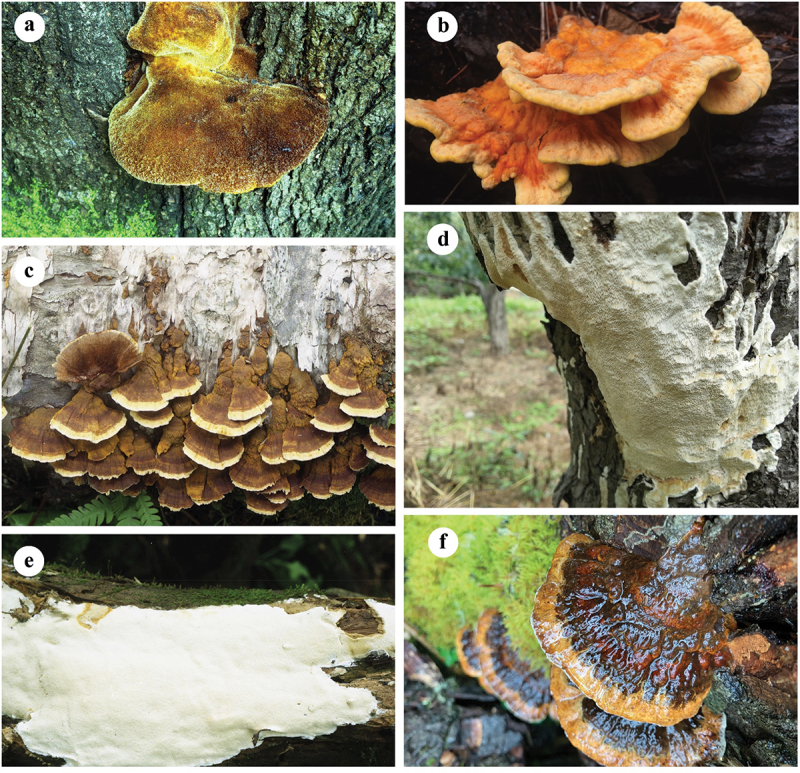
Basidiomata of some typical forest pathogens. (a) *Inonotus hispidus*. (b) *Laetiporus montanus*. (c) *Mensularia radiata*. (d) *Perenniporia pyricola*. (e) *Perenniporia subacida*. (f) *Pseudoinonotus tibeticus*.

**Figure 3.**
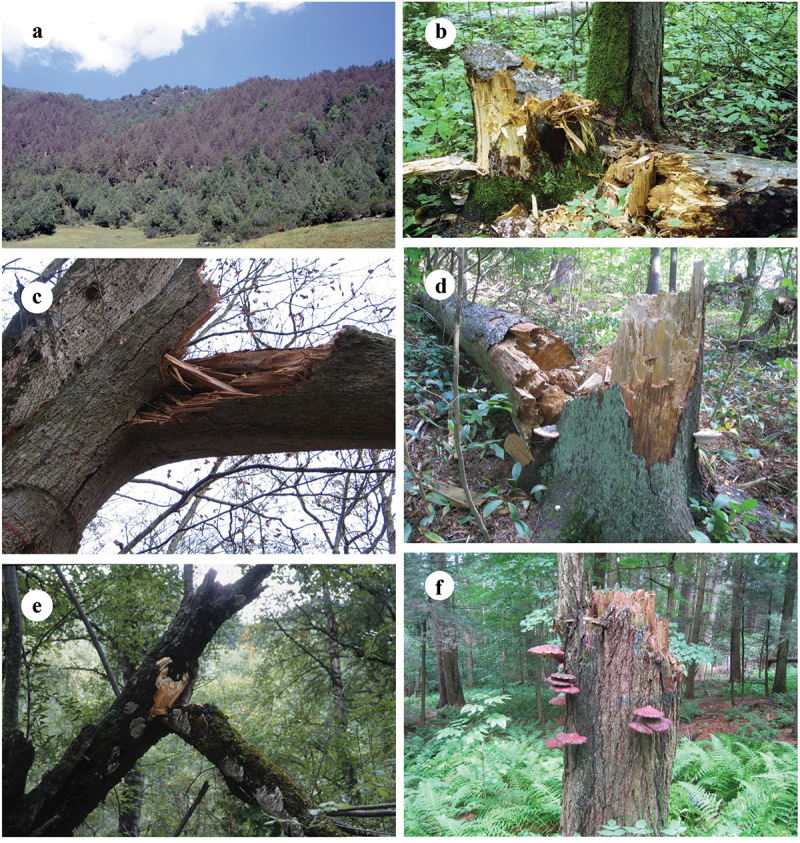
Symptoms of some typical forest pathogens. (a) *Coniferiporia qilianensis*. (b) *Coniferiporia sulphurascens*. (c) *Fomes fomentarius*. (d) *Fomitiopsis subpinicola*. (e) *Funalia trogii*. (f) *Ganoderma tsugae*.

**Figure 4.**
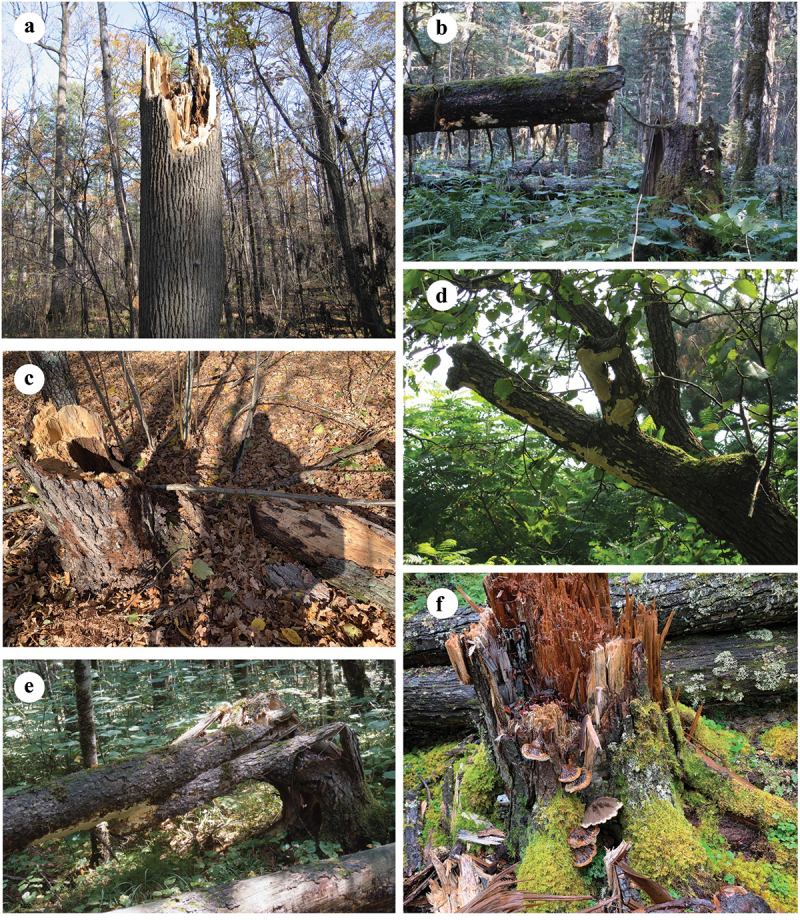
Symptoms of some typical forest pathogens. (a) *Inonotus hispidus*. (b) *Laetiporus montanus*. (c) *Mensularia radiata*. (d) *Perenniporia pyricola*. (e) *Perenniporia subacida*. (f) *Pseudoinonotus tibeticus*.

The host(s), type of forest, rot type, damage caused, and distribution are listed after each species. The host trees are arranged according to the fungal occurring frequency on them, and the distribution is arranged alphabetically by the Chinese provinces. Among them, 72 species are recorded as pathogenic fungi in China for the first time, and they are marked with an asterisk (*) before the names. Four plates (Figures 1–4) show typical basidiomata and symptoms for 12 species in the forests.

***Abundisporus quercicola*** **Y.C. Dai**, polypore, Polyporaceae, Polyporales

Mostly on *Quercus*, especially on *Quercus semicar-pifolia* in native forests.

White rot, causes butt rot.

Northern Yunnan.

***Armillaria ostoyae*** **(Romagn.) Herink**, agaric, Physalacriaceae, Agaricales

Mostly on *Larix*, *Pinus*, *Abies,* and *Picea*, but frequently on many angiosperm trees in both native forests and plantations.

White rot, causes root rot.

Beijing, Heilongjiang, Inner Mongolia, Jilin, Liaoning, and Ningxia.

***Athelia scutellaris*** **(Berk. & M.A. Curtis) Gilb**., corticioid, Atheliaceae, Atheliales

Mainly on species of *Camellia* in plantations.

White rot, causes butt rot.

Anhui, Guangdong, Guangxi, Hunan, and Jiangxi.

***Baltazaria galactina*** **(Fr.) Leal-Dutra, Dentinger & G.W. Griff**, corticioid, Peniophoraceae, Russulales

On many angiosperm trees in native forests.

White rot, causes butt and root rot.

Northeast China.

***Bjerkandera adusta*** **(Willd.) P. Karst**., polypore, Phanerochaetaceae, Polyporales

Mostly on *Populus*, *Betula*, *Tilia*, *Quercus*, and *Salix* in both native forests and plantations.

White rot, causes butt rot.

Temperate to subtropical China.

*Bondarzewia dickinsii **(Berk.) Jia J. Chen, B.K. Cui & Y.C. Dai**, polypore, Bondarzewiaceae, Russulales

Mostly on *Quercus* and *Castanea* in native forests.

White rot, causes root rot.

Anhui, Guangdong, and Jiangxi.

***Bondarzewia podocarpi*** **Y.C. Dai & B.K. Cui**, polypore, Bondarzewiaceae, Russulales

On *Dacrydium pierrei* and *Podocarpus imbricatus* in native forests.

White rot, causes root rot.

Hainan.

*Bondarzewia submesenterica **Jia J. Chen, B.K. Cui & Y.C. Dai**, polypore, Bondarzewiaceae, Russulales

On *Abies* and *Pinus* in native forests.

White rot, causes root rot.

Sichuan and Yunnan.

*Bondarzewia tibetica **B.K. Cui, J. Song & Jia J. Chen**, polypore, Bondarzewiaceae, Russulales

On *Picea* in native forests.

White rot, causes root rot.

Tibet.

***Bridgeoporus sinensis*** **(X.L. Zeng) F. Wu, Jia J. Chen & Y.C. Dai**, polypore, Schizoporaceae, Hymenochaetales

Exclusively on *Populus ussuriensis* in old-growth native forests.

White rot, causes root rot.

Heilongjiang and Jilin.

***Cerrena unicolor*** **(Bull.) Murrill**, hydnoid, Cerrenaceae, Polyporales

On *Betula*, *Acer*, *Populus*, *Salix*, *Quercus*, and other angiosperm trees in native forests, plantations, and gardens.

White rot, causes sapwood rot.

Beijing, Gansu, Hebei, Heilongjiang, Henan, Hubei, Inner Mongolia, Jiangsu, Jilin, Liaoning, Ningxia, Qinghai, Shandong, Shanxi, Tianjin, Tibet, and Yunnan.

***Cerrena zonata*** **(Berk.) H.S. Yuan**, hydnoid, Cerrenaceae, Polyporales

On many angiosperm trees in native forests, plantations, and gardens.

White rot, causes butt and root rot.

Anhui, Fujian, Guangdong, Guangxi, Guizhou, Hainan, Hubei, Hunan, Jiangsu, Shaanxi, Sichuan, Yunnan, and Zhejiang.

***Chondrostereum purpureum*** **(Pers.) Pouzar**, corticioid, Cyphellaceae, Agaricales

On many angiosperm trees, especially on *Betula* and *Prunus*, in native forests, plantations, and gardens.

White rot, causes butt and root rot.

Northeast and northwest China, and mountainous areas of subtropical China.

***Climacocystis borealis*** **(Fr.) Kotl. & Pouzar**, polypore, Fomitopsidaceae, Polyporales

Mostly on *Picea*, rarely on *Pinus* in native forests.

White rot, causes butt rot.

Heilongjiang and Jilin.

*Climacocystis montana **B.K. Cui & J. Song**, polypore, Fomitopsidaceae, Polyporales

On *Picea* in native forests.

White rot, causes butt rot.

Sichuan, Tibet, and Yunnan.

***Climacodon septentrionalis*** **(Fr.) P. Karst**., hydnoid, Meruliaceae, Polyporales

Mostly on *Acer* in both native forests and gardens.

White rot, causes heartwood rot.

Heilongjiang and Jilin.

***Coniferiporia qilianensis*** **(B.K. Cui, L.W. Zhou & Y.C. Dai) L.W. Zhou & Y.C. Dai**, polypore, Hymenochaetaceae, Hymenochaetales

Exclusively on *Sabina przewalskii* in native forests.

White rot, causes root rot.

Qinghai.

***Coniferiporia sulphurascens*** **(Pilát) L.W. Zhou & Y.C. Dai**, polypore, Hymenochaetaceae, Hymenochaetales

Mainly on *Picea*, *Pinus*, *Abies*, and *Larix* in old growth native forests.

White rot, causes root rot.

Heilongjiang, Inner Mongolia, Jilin, Sichuan, Tibet, Xinjiang, and Yunnan.

***Daedalea incana*** **(P. Karst.) Sacc. & D. Sacc**., polypore, Fomitopsidaceae, Polyporales

On many angiosperm trees in native forests.

Brown rot, causes heartwood rot.

Tropical China.

***Daedaleopsis confragosa*** **(Bolton) J. Schröt**., polypore, Polyporaceae, Polyporales

Mostly on *Salix* and occasionally on other angios-perm trees in both native forests and plantations.

White rot, causes butt rot.

Beijing, Gansu, Hebei, Heilongjiang, Henan, Hubei, Inner Mongolia, Jiangsu, Jiangxi, Jilin, Liaoning, Ningxia, Qinghai, Shaanxi, Sichuan, Tianjin, Tibet, Xinjiang, and Yunnan.

***Daedaleopsis sinensis*** **(Lloyd) Y.C. Dai**, polypore, Polyporaceae, Polyporales

Mostly on *Betula* and *Alnus* in native forests.

White rot, causes heartwood rot.

Heilongjiang and Jilin.

***Desarmillaria tabescens*** **(Scop.) R.A. Koch & Aime**, agaric, Physalacriaceae, Agaricales

Mostly on *Populus* and *Salix*, sometimes on *Malus*, *Prunus,* and *Ziziphus* in plantations and gardens.

White rot, causes root rot.

Beijing, Hebei, Ningxia, Shandong, and Tianjin.

*Flammulina filiformis **(Z.W. Ge, X.B. Liu & Zhu L. Yang) P.M. Wang, Y.C. Dai, E. Horak & Zhu L. Yang**, agaric, Physalacriaceae, Agaricales

Mostly on *Salix*, also on other angiosperm trees in native forests and plantations.

White rot, causes butt rot.

Chongqing, Gansu, Guizhou, Henan, Hubei, Hunan, Inner Mongolia, Shanxi, Sichuan, and Yunnan.

*Flammulina rossica **Redhead & R.H. Petersen**, agaric, Physalacriaceae, Agaricales

Mostly on *Salix*, also on other angiosperm trees in native forests and plantations.

White rot, causes butt rot.

Jilin, Shanxi, Sichuan, Tibet, and Yunnan.

***Fomes fomentarius*** **(L.) Fr**., polypore, Polyporaceae, Polyporales

Commonly on *Betula* and *Salix*, and also on other angiosperm trees in both native forests and plantations.

White rot, causes heartwood rot.

Boreal and temperate China, and mountain areas of subtropical China.

***Fomitiporella inermis*** **(Ellis & Everh.) Murrill**, polypore, Hymenochaetaceae, Hymenochaetales

On many angiosperm trees in native forests, plantations, and gardens.

White rot, causes heartwood rot.

Subtropical to tropical China.

*Fomitiporia alpina **B.K. Cui & Hong Chen**, polypore, Hymenochaetaceae, Hymenochaetaceae

On *Abies* in old growth native forests.

White rot, causes heartwood rot.

Sichuan, Tibet, and Yunnan.

***Fomitiporia bannaensis*** **Y.C. Dai**, polypore, Hymenochaetaceae, Hymenochaetaceae

On many angiosperm trees in native forests.

White rot, causes heartwood rot.

Subtropical to tropical China.

*Fomitiporia carpinea **X.H. Ji, X.M. Tian & Y.C. Dai**, polypore, Hymenochaetaceae, Hymenochaetaceae

On *Carpinus* in native forests.

White rot, causes heartwood rot.

Gansu.

*Fomitiporia gaoligongensis **B.K. Cui & Hong Chen**, polypore, Hymenochaetaceae, Hymenochaetaceae

On angiosperm tree species in native forests.

White rot, causes heartwood rot.

Northern Yunnan.

***Fomitiporia hartigii*** **(Allesch. & Schnabl) Fiasson & Niemelä**, polypore, Hymenochaetaceae, Hymenochaetaceae

On *Abies*, and occasionally on other trees of Pinaceae in old growth native forests.

White rot, causes heartwood rot.

Heilongjiang and Jilin.

*Fomitiporia lagerstroemiae **X.H. Ji, X.M. Tian & Y.C. Dai**, polypore, Hymenochaetaceae, Hymenochaetaceae

On *Cryptocarya* and *Quercus* in native forests and gardens.

White rot, causes heartwood rot.

Beijing and Sichuan.

*Fomitiporia norbulingka **B.K. Cui & Hong Chen**, polypore, Hymenochaetaceae, Hymenochaetaceae

On *Hippophae* in native forests and gardens.

White rot, causes heartwood rot.

Sichuan, Tibet, and Yunnan.

*Fomitiporia pentaphylacis **L.W. Zhou**, polypore, Hymenochaetaceae, Hymenochaetaceae

On *Pentaphylax* in native forests.

White rot, causes heartwood rot.

Guangxi and Hainan.

***Fomitiporia punctata*** **(P. Karst.) Murrill**, polypore, Hymenochaetaceae, Hymenochaetaceae

On many angiosperm tree species in native forests, plantations, and gardens.

White rot, causes heartwood rot.

Boreal and temperate China, and mountainous areas of subtropical China.

***Fomitiporia punicata*** **Y.C. Dai, B.K. Cui & Decock**, polypore, Hymenochaetaceae, Hymenochaetaceae

Mainly on *Punica granatum* and *Sophora japonica*, occasionally on other angiosperm tree species in plantations and gardens.

White rot, causes heartwood rot.

Beijing, Chongqing, Hebei, Ningxia, Shanxi, Sichuan, and Xinjiang.

***Fomitiporia pusilla*** **(Lloyd) Y.C. Dai**, polypore, Hymenochaetaceae, Hymenochaetaceae

On angiosperm trees in native forests.

White rot, causes heartwood rot.

Tropical China.

*Fomitiporia rhamnoides **T.Z. Liu & F. Wu**, polypore, Hymenochaetaceae, Hymenochaetaceae

On *Hippophae* and *Elaeagnus* in native forests and plantations.

White rot, causes heartwood rot.

Beijing, Gansu, Hebei, Qinghai, Shanxi, and Tibet.

*Fomitiporia subhippophaëicola **B.K. Cui & H. Chen**, polypore, Hymenochaetaceae, Hymenochaetaceae

On *Hippophae* in native forests.

White rot, causes heartwood rot.

Tibet.

*Fomitiporia subtropica **B.K. Cui & Hong Chen**, polypore, Hymenochaetaceae, Hymenochaetaceae

On angiosperm tree species in native forests.

White rot, causes heartwood rot.

Guangdong.

***Fomitiporia torreyae*** **Y.C. Dai & B.K. Cui**, polypore, Hymenochaetaceae, Hymenochaetaceae

On *Cryptomeria*, *Cunninghamia,* and *Torreya grandis*, also on many angiosperm tree species, e.g. *Cinnamomum*, *Cyclobalanopsis*, *Morus*, *Puranta*, *Pasitenia*, *Rhododendron*, and *Vitex* in native forests.

White rot, causes heartwood rot.

Anhui, Beijing, Fujian, Guangxi, Guizhou, Hubei, Hunan, Jiangxi, Shanxi, and Tianjin.

*Fomitopsis abieticola **B.K. Cui, M.L. Han & Shun Liu**, polypore, Fomitopsidaceae, Polyporales

Mostly on *Abies* in old growth native forests.

Brown rot, causes heartwood rot.

Northern Yunnan.

***Fomitopsis betulina*** **(Bull.) B.K. Cui, M.L. Han & Y.C. Dai**, polypore, Fomitopsidaceae, Polyporales

Exclusively on *Betula* mostly in native forests, rarely in plantations.

Brown rot, causes heartwood rot.

Boreal and temperate China.

***Fomitopsis bondartsevae*** **(Spirin) A.M.S. Soares & Gibertoni**, polypore, Fomitopsidaceae, Polyporales

Mostly on *Prunus*, especially on *Prunus persica* in plantations.

Brown rot, causes butt rot.

Northern China.

*Fomitopsis hengduanensis **B.K. Cui & Shun Liu**, polypore, Fomitopsidaceae, Polyporales

Mostly on *Picea* and *Abies* in old-growth native forests.

Brown rot, causes heartwood rot.

Northern Yunnan and western Sichuan.

*Fomitopsis massoniana **B.K. Cui, M.L. Han & Shun Liu**, polypore, Fomitopsidaceae, Polyporales

Mostly on *Pinus massoniana* in old-growth native forests.

Brown rot, causes heartwood rot.

Fujian, Guangdong, and Zhejiang.

***Fomitopsis nivosa*** **(Berk.) Gilb. & Ryvarden**, polypore, Fomitopsidaceae, Polyporales

On *Cinnamomum* in gardens.

Brown rot, causes heartwood rot.

Guangxi and Sichuan.

*Fomitopsis palustri**s (Berk. & M.A. Curtis) Gilb. & Ryvarden**, polypore, Fomitopsidaceae, Polyporales

On *Fraxinus*, *Prunus,* and other angiosperm tree species in gardens.

Brown rot, causes butt and root rot.

Beijing, Guangdong, Hainan, and Sichuan.

*Fomitopsis subpinicola **B.K. Cui, M.L. Han & Shun Liu**, polypore, Fomitopsidaceae, Polyporales

Mostly on *Larix*, *Picea,* and *Pinus*, but also on some angiosperm tree species, e.g. *Betula* and *Populus* in native forests.

Brown rot, causes heartwood rot.

Heilongjiang, Jilin, and Liaoning.

*Fomitopsis tianshanensis **B.K. Cui & Shun Liu**, polypore, Fomitopsidaceae, Polyporales

Mostly on *Picea* in old-growth native forests.

Brown rot, causes heartwood rot.

Xinjiang.

***Fulvifomes durissimus*** **(Lloyd) Bondartseva & S. Herrera**, polypore, Hymenochaetaceae, Hymenochaetales

On angiosperm tree species in native forests.

White rot, causes heartwood rot.

Hainan and tropical Yunnan.

***Fulvifomes lloydii*** **(Cleland) Y.C. Dai & X.H. Ji**, polypore, Hymenochaetaceae, Hymenochaetales

On many angiosperm tree species in native forests.

White rot, causes butt and root rot.

Hainan.

***Fulvifomes mcgregorii*** **(Bres.) Y.C. Dai**, polypore, Hymenochaetaceae, Hymenochaetales

Mostly on *Acer* and *Quercus* in native forests.

White rot, causes heartwood rot.

Yunnan.

***Funalia trogii*** **(Berk.) Bondartsev & Singer**, polypore, Polyporaceae, Polyporales

Commonly on *Populus* and *Salix* in native forests, plantations, and gardens.

White rot, causes heartwood rot.

Boreal, temperate, and subtropical China.

***Fuscoporia gilva*** **(Schwein.) T. Wagner & M. Fisch**., polypore, Hymenochaetaceae, Hymenochaetales

Mostly on *Castanopsis* in native forests, also grows on other angiosperm tree species, but causes damage on *Castanopsis* only.

White rot, causes heartwood rot.

Anhui, Fujian, Guangdong, Guangxi, Hainan, and Sichuan.

***Fuscoporia rhabarbarina*** **(Berk.) Groposo, Log.-Leite & Góes-Neto**, polypore, Hymenochaetaceae, Hymenochaetales

On many angiosperm tree species in native forests.

White rot, causes heartwood rot.

Fujian, Guangdong, Guangxi, Guizhou, Hainan, Hebei, Hubei, Jiangxi, Sichuan, Taiwan Province, and Yunnan.

***Fuscoporia torulosa*** **(Pers.) T. Wagner & M. Fisch**., polypore, Hymenochaetaceae, Hymenochaetales

Mostly on angiosperm tree species, occasionally on *Pinus* in native forests and plantations.

White rot and heartwood rot.

Beijing, Guangxi, Hainan, Henan, Hunan, Sichuan, Tianjin, and Yunnan.

*Ganoderma angustisporum **J.H. Xing, B.K. Cui & Y.C. Dai**, polypore, Ganodermataceae, Polyporales

On *Casuarina equisetifolia* in plantations.

White rot, causes butt and root rot.

Fujian, Guangdong, Guangxi, and Yunnan.

***Ganoderma applanatum*** **(Pers.) Pat**., polypore, Ganodermataceae, Polyporales

On many angiosperm tree species in native forests, plantations, and gardens.

White rot, causes butt rot.

Boreal to temperate China.

***Ganoderma australe*** **(Fr.) Pat**., polypore, Ganodermataceae, Polyporales

On many angiosperm tree species in native forests, plantations, and gardens.

White rot, causes butt and root rot.

Subtropical to tropical China.

*Ganoderma casuarinicola **J.H. Xing, B.K. Cui & Y.C. Dai**, polypore, Ganodermataceae, Polyporales

On *Casuarina equisetifolia* in plantations.

White rot, causes butt and root rot.

Guangdong and Hainan.

***Ganoderma multipileum*** **Ding Hou**, polypore, Ganodermataceae, Polyporales

On many angiosperm tree species, especially on *Delonix* in plantations and gardens.

White rot, causes butt and root rot.

Guangdong, Hainan, Sichuan, Taiwan Province, and Yunnan.

*Ganoderma mutabile **Y. Cao & H.S. Yuan**, polypore, Ganodermataceae, Polyporales

On several angiosperm tree species, e.g. *Symplocos sumuntia* in native forests.

White rot, causes butt rot.

Yunnan.

***Ganoderma philippii*** **(Bres. & Henn. ex Sacc.) Bres**., polypore, Ganodermataceae, Polyporales

On *Hevea*, *Schefflera*, *Lannea*, *Melia*, *Sapindus*, *Vernicia,* and *Coffea*, especially in plantations of *Hevea brasiliensis*.

White rot, causes root rot.

Fujian, Guangdong, Guangxi, Hainan, and Yunnan.

***Ganoderma tropicum*** **(Jungh.) Bres**., polypore, Ganodermataceae, Polyporales

On several species of *Acacia* in plantations and gardens, especially on *Acacia richii*.

White rot, causes butt and root rot.

Fujian, Guangdong, Guangxi, Hainan, Taiwan Province, and Yunnan.

***Ganoderma tsugae*** **Murrill**, polypore, Ganodermataceae, Polyporales

Mostly on *Larix* in old growth native forests.

White rot, causes butt rot.

Heilongjiang, Inner Mongolia, and Jilin.

***Ganoderma weberianum*** **(Bres. & Henn. ex Sacc.) Steyaert**, polypore, Ganodermataceae, Polyporales

On many angiosperm tree species, especially on *Ficus* in native forests and plantations.

White rot, causes butt and root rot.

Guangdong, Guangxi, and Yunnan.

***Ganoderma williamsianum*** **Murrill**, polypore, Ganodermataceae, Polyporales

On many angiosperm tree species in native forests.

White rot, causes butt and root rot.

Tropical Yunnan.

***Gyrodontium sacchari*** **(Spreng.) Hjortstam**, hydnoid, Coniophoraceae, Boletales

On angiosperm tree species in native forests.

Brown rot, causes root rot.

Guangdong, Guangxi, Hainan, and Yunnan.

***Haploporus odorus*** **(Sommerf.) Bondartsev & Singer**, polypore, Polyporaceae, Polyporales

Mainly on *Salix* and occasionally on *Populus* and *Syringa* in native forests.

White rot, causes heartwood rot.

Hubei, Jilin, Ningxia, Shaanxi, Shanxi, Sichuan, Tibet, and Yunnan.

***Helicobasidium purpureum*** **(Tul.) Pat**., corticioid, Helicobasidiaceae, Helicobasidiales

Mainly on *Hevea, Mangifera, Camellia, Populus,* and *Melia* in plantations.

White rot, causes root rot.

Shaanxi and Yunnan.

***Hericium erinaceus*** **(Bull.) Pers**., hydnoid, Hericiaceae, Russulales

Mostly on *Quercus mongolica* in native forests.

White rot, causes heartwood rot.

Beijing, Heilongjiang, Inner Mongolia, Jilin, Liaoning, and Sichuan.

***Hornodermoporus latissimus*** **(Bres.) B.K. Cui & Y.C. Dai**, polypore, Polyporaceae, Polyporales

On angiosperm tree species, e.g. *Gmelina*, in native forests.

White rot, causes heartwood rot.

Hainan.

***Hornodermoporus martius*** **(Berk.) Teixeira**, polypore, Polyporaceae, Polyporales

On angiosperm tree species in native forests and gardens.

White rot, causes heartwood rot.

Fujian, Guangdong, Guangxi, and Yunnan.

***Hymenochaete microcycla*** **(Zipp. ex Lév.) Spirin & Miettinen**, polypore, Hymenochaetaceae, Hymenochaetales

On many angiosperm tree species in native forests and plantations.

White rot, causes root rot.

Subtropical to tropical China.

***Hypsizygus ulmarius*** **(Bull.) Redhead**, agaric, Lyophyllaceae, Agaricales

Mostly on *Betula* and *Ulmus* in native forests.

White rot, causes heartwood rot.

Heilongjiang and Jilin.

***Inocutis levis*** **(P. Karst.) Y.C. Dai**, polypore, Hymenochaetaceae, Hymenochaetales

Mostly on *Populus* and occasionally on *Ulmus* in native forests, plantations, and gardens.

White rot, causes heartwood rot.

Ningxia and Xinjiang.

*Inocutis rheades **(Pers.) Fiasson & Niemelä**, polypore, Hymenochaetaceae, Hymenochaetales

Mainly on *Populus* in native forests and plantations.

White rot, causes butt rot.

Beijing and Jilin.

***Inocutis subdryophila*** **Y.C. Dai & H.S. Yuan**, polypore, Hymenochaetaceae, Hymenochaetales

On angiosperm tree species in native forests.

White rot, causes heartwood rot.

Tibet.

***Inocutis tamaricis*** **(Pat.) Fiasson & Niemelä**, polypore, Hymenochaetaceae, Hymenochaetales

Exclusively on *Tamarix chinensis* in plantations and gardens.

White rot, causes heartwood rot.

Beijing, Hebei, Jiangsu, Shaanxi, and Shandong.

*Inonotus casuarinae **L.S. Bian**, polypore, Hymenochaetaceae, Hymenochaetales

Exclusively on *Casuarina equisetifolia* in plantations.

White rot, causes heartwood rot.

Hainan.

***Inonotus compositus*** **Han C. Wang**, polypore, Hymenochaetaceae, Hymenochaetales

Exclusively on *Quercus* in native forests.

White rot, causes heartwood rot.

Tibet.

***Inonotus diverticuloseta*** **Pegler**, polypore, Hymenochaetaceae, Hymenochaetales

On *Quercus, Ulmus* and *Platanus* in plantations and gardens.

White rot, causes heartwood rot.

Beijing, Jiangsu, and Zhejiang.

***Inonotus hispidus*** **(Bull.) P. Karst**., polypore, Hymenochaetaceae, Hymenochaetales

On many angiosperm tree species, especially on *Fraxinus*, *Malus,* and *Ulmus* in native forests, plantations, and gardens.

White rot, causes heartwood rot.

Temperate China.

***Inonotus krawtzewii*** **(Pilát) Pilát**, polypore, Hymenochaetaceae, Hymenochaetales

On *Quercus* and *Populus* in native forests.

White rot, causes canker rot.

Heilongjiang, Inner Mongolia, and Shaanxi.

***Inonotus obliquus*** **(Fr.) Pilát**, polypore, Hymenochaetaceae, Hymenochaetales

Exclusively on *Betula*, especially on *B. platyphylla* and *B. ermanii* in native forests.

White rot, causes sapwood and canker rot.

Beijing, Gansu, Hebei, Heilongjiang, Inner Mongolia, Jilin, Shaanxi, Shanxi, and Xinjiang.

***Inonotus ochroporus*** **(Van der Byl) Pegler**, polypore, Hymenochaetaceae, Hymenochaetales

On angiosperm tree species in native forests.

White rot, causes heartwood rot.

Southern Yunnan.

***Inonotus plorans*** **(Pat.) Bondartsev & Singer**, polypore, Hymenochaetaceae, Hymenoch-aetales

Mostly on *Populus* and *Ziziphus* in plantations and gardens.

White rot, causes heartwood rot.

Ningxia and Xinjiang.

***Inonotus rickii*** **(Pat.) D.A. Reid**, polypore, Hymenochaetaceae, Hymenochaetales

On many angiosperm tree species, especially on *Hevea brasiliensis* in plantations and gardens.

White rot, causes heartwood rot.

Fujian, Hainan, and Sichuan.

***Irpex laceratus*** **(N. Maek., Suhara & R. Kondo) C.C. Chen & Sheng H. Wu**, polypore, Irpicaceae, Polyporales

On many angiosperm tree species in native forests and plantations.

White rot, causes butt and root rot.

Warm temperate to subtropical China.

*Laetiporus ailaoshanensis **B.K. Cui & J. Song**, polypore, Laetiporaceae, Polyporales

Mostly on *Lithocarpus* in native forests.

Brown rot, causes heartwood rot.

Yunnan.

***Laetiporus cremeiporus*** **Y. Ota & T. Hatt**., polypore, Laetiporaceae, Polyporales

Mostly on *Quercus*, rarely on other angiosperm tree species in native forests.

Brown rot, causes heartwood rot.

Gansu, Heilongjiang, Henan, Hubei, Hunan, Jilin, Liaoning, Shaanxi, Shanxi, and Sichuan.

*Laetiporus medogensis **J. Song & B.K. Cui**, polypore, Laetiporaceae, Polyporales

Mostly on *Abies* in native forests.

Brown rot, causes heartwood rot.

Tibet.

***Laetiporus montanus*** **Černý ex Tomšovský & Jankovský**, polypore, Laetiporaceae, Polyporales

Mostly on *Larix*, occasionally on *Picea* in native forests.

Brown rot, causes heartwood rot.

Gansu, Heilongjiang, Inner Mongolia, Jilin, Xinjiang, and Yunnan.

***Laetiporus sulphureus*** **(Bull.) Murrill**, polypore, Laetiporaceae, Polyporales

Mostly on *Salix* in plantations and gardens.

Brown rot, causes heartwood rot.

Xinjiang.

***Laetiporus versisporus*** **(Lloyd) Imazeki**, polypore, Laetiporaceae, Polyporales

Mostly on *Cyclobalanopsis* in native forests.

Brown rot, causes heartwood rot.

Fujian, Hainan, Jiangxi, Yunnan, and Zhejiang.

*Laetiporus xinjiangensis **J. Song, Y.C. Dai & B.K. Cui**, polypore, Laetiporaceae, Polyporales

Mostly on *Betula* in native forests.

Brown rot, causes heartwood rot.

Xinjiang.

*Laetiporus zonatus **B.K. Cui & J. Song**, polypore, Laetiporaceae, Polyporales

On several angiosperm and gymnosperm tree species, e.g. *Abies*, *Picea*, *Castanopsis*, *Hippopha*e and *Quercus* in native forests.

Brown rot, causes heartwood rot.

Sichuan and Yunnan.

***Laricifomes officinalis*** **(Vill.) Kotl. & Pouzar**, polypore, Fomitopsidaceae, Polyporales

On *Larix*, and occasionally on *Pinus*, *Picea,* and *Abies* in old growth native forests.

Brown rot, causes heartwood rot.

Heilongjiang, Inner Mongolia, Jilin, Sichuan, and Xinjiang.

***Lenzitopsis daii*** **L.W. Zhou & Kõljalg**, polypore, Thelephoraceae, Thelephorales

Mostly on *Sabina* in plantations and gardens.

White rot, causes heartwood rot.

Beijing and Sichuan.

***Leucophellinus irpicoides*** **(Bondartsev ex Pilát) Bondartsev & Singer**, polypore, Schizoporaceae, Hymenochaetales

Mostly on *Acer*, occasionally on *Populus* in native forests.

White rot, causes heartwood rot.

Beijing, Heilongjiang, Henan, Jilin, Liaoning, Shaanxi, Shanxi, Yunnan, and Zhejiang.

***Melanoporia castanea*** **(Imazeki) T. Hatt. & Ryvarden**, polypore, Nigrofomitaceae, Hymenochaetales

Exclusively on *Quercus mongolica* in old growth native forests.

Brown rot, causes root rot.

Heilongjiang, Inner Mongolia, Jilin, and Liaoning.

***Mensularia radiata*** **(Sowerby) Lázaro Ibiza**, polypore, Hymenochaetaceae, Hymenocha-etales

Mostly on *Betula*, occasionally on *Alnus* and *Populus* in native forests and plantations.

White rot, causes butt and root rot.

Beijing, Chongqing, Gansu, Hebei, Heilongjiang, Inner Mongolia, Jilin, Liaoning,Qinghai, Shanxi, Sichuan, Tibet, and Yunnan.

***Necator salmonicolor*** **(Berk. & Broome) K.H. Larss., Redhead & T.W. May**, corticioid, Corticiaceae, Corticiales

Mostly on *Camellia*, *Hevea*, *Citrus*, *Malus*, and *Mangifera* in plantations.

White rot, causes butt and root rot.

Guangdong, Guangxi, Jiangxi, Sichuan, and Zhejiang.

***Neomensularia kanehirae*** **(Yasuda) F. Wu, L.W. Zhou & Y.C. Dai**, polypore, Hymenochaetaceae, Hymenochaetales

On many angiosperm tree species, e.g. *Schima*, in native forests, plantations, and gardens.

White rot, causes heartwood rot.

Fujian, Guangdong, Hainan, Jiangsu, Jiangxi, and Yunnan.

***Niveoporofomes spraguei*** **(Berk. & M.A. Curtis) B.K. Cui, M.L. Han & Y.C. Dai**, polypore, Fomitopsidaceae, Polyporales

On *Castanea*, *Castanopsis*, and *Cyclobalanopsis* in nature forests and plantations.

Brown rot, causes heartwood rot.

Fujian, Guangdong, Guangxi, Hainan, Hubei, Hunan, Jiangxi, and Yunnan.

***Ochrosporellus pachyphloeus*** **(Pat.) Y.C. Dai & F. Wu**, polypore, Hymenochaetaceae, Hymenochaetales

On angiosperm tree species in native forests.

White rot, causes heartwood rot.

Hainan and tropical Yunnan.

*Onnia himalayana **Y.C. Dai, H. Zhao & Meng Zhou**, polypore, Hymenochaetaceae, Hymenochaetales

On *Pinus yunnanensis* in native forests.

White rot, causes root rot.

Yunnan.

***Onnia leporina*** **(Fr.) H. Jahn**, polypore, Hymenochaetaceae, Hymenochaetales

Mostly on *Picea* in native forests.

White rot, causes root rot.

Hebei, Heilongjiang, and Jilin.

*Onnia microspora **Y.C. Dai & L.W. Zhou**, polypore, Hymenochaetaceae, Hymenochaetales

On *Pinus massoniana* in native forests.

White rot, causes root rot.

Anhui, Jiangxi, and Zhejiang.

*Onnia tibetica **Y.C. Dai & S.H. He**, polypore, Hymenochaetaceae, Hymenochaetales

On *Pinus yunnanensis* in native forests.

White rot, causes root rot.

Tibet.

***Onnia tomentosa*** **(Fr.) P. Karst**., polypore, Hymenochaetaceae, Hymenochaetales

On *Abies*, *Larix*, *Picea,* and *Pinus* in native forests.

White rot, causes root rot.

Inner Mongolia, Jilin, Sichuan, and Yunnan.

***Parmastomyces taxi*** **(Bondartsev) Y.C. Dai & Niemelä**, polypore, Fomitopsidaceae, Polyporales

Exclusively on *Larix olgensis* in old growth native forests.

Brown rot, causes heartwood rot.

Jilin.

***Perenniporia pyricola*** **Y.C. Dai & B.K. Cui**, polypore, Polyporaceae, Polyporales

Mostly on *Pyrus* and *Prunus* in plantations and gardens.

White rot, causes heartwood rot.

Beijing, Hebei, Liaoning, and Tianjin.

***Perenniporia subacida*** **(Peck) Donk**, polypore, Polyporaceae, Polyporales

On *Picea*, *Abies*, and *Larix* in old growth native forests.

White rot, causes butt and root rot.

Heilongjiang, Jilin, and Yunnan.

***Perenniporia truncatospora*** **(Lloyd) Ryvarden**, polypore, Polyporaceae, Polyporales

On angiosperm tree species, e.g. *Platycarya*, *Pyrus*, and *Quercus*, in plantations and gardens.

White rot, causes heartwood rot.

Beijing, Liaoning, Sichuan, Tianjin, and Yunnan.

*Phaeolus asiae-orientalis **Y.C. Dai & Yuan Yuan**, polypore, Laetiporaceae, Polyporales

On *Pinus*, *Larix*, *Picea*, and *Abies* in old growth native forests.

Brown rot, causes butt and root rot.

Heilongjiang, Inner Mongolia, and Jilin.

*Phaeolus yunnanensis **Y.C. Dai & Yuan Yuan**, polypore, Laetiporaceae, Polyporales

On *Pinus yunnanensis* in native forests and plantations.

Brown rot, causes butt and root rot.

Yunnan.

***Phellinopsis conchata*** **(Pers.) Y.C. Dai**, polypore, Hymenochaetaceae, Hymenochaetales

Mainly on *Salix*, occasionally on other angiosperm tree species in native forests, but occasionally in plantations and gardens.

White rot, causes heartwood rot.

Gansu, Hebei, Henan, Jilin, Qinghai, Tibet, and Yunnan.

*Phellinopsis lonicericola **L.W. Zhou**, polypore, Hymenochaetaceae, Hymenochaetales

Mainly on *Lonicera* in native forests.

White rot, causes heartwood rot.

Gansu, northern Yunnan, Sichuan, and Tibet.

***Phellinus alni*** **(Bondartsev) Parmasto**, polypore, Hymenochaetaceae, Hymenochaetales

Mostly on *Alnus* in native forests.

White rot, causes heartwood rot.

Heilongjiang, Inner Mongolia, Jilin, Tibet, and Yunnan.

***Phellinus igniarius*** **(L.) Quél**., polypore, Hymenochaetaceae, Hymenochaetales

On many angiosperm tree species, especially on *Salix*, in native forests, plantations, and gardens.

White rot, causes heartwood rot.

Boreal and temperate China.

*Phellinus mori **Y.C. Dai & B.K. Cui**, polypore, Hymenochaetaceae, Hymenochaetales

Exclusively on *Morus* in native forests, plantations, and gardens.

White rot, causes heartwood rot.

Beijing and Heilongjiang.

*Phellinus nigricans **(Fr.) P. Karst**., polypore, Hymenochaetaceae, Hymenochaetales

Mostly on *Betula* and *Alnus* in native forests.

White rot, causes heartwood rot.

Boreal and temperate China.

*Phellinus orientoasiaticus **L.W. Zhou & Y.C. Dai**, polypore, Hymenochaetaceae, Hymenochaetales

Mostly on *Prunus* in native forests, plantations, and gardens.

White rot, causes heartwood rot.

Temperate China.

*Phellinus padicola **L.W. Zhou & Y.C. Dai**, polypore, Hymenochaetaceae, Hymenochaetales

Mostly on *Padus* in native forests.

White rot, causes heartwood rot.

Sichuan and Tibet.

*Phellinus parmastoi **L.W. Zhou & Y.C. Dai**, polypore, Hymenochaetaceae, Hymenochaetales

Almost exclusively on *Betula* in native forests.

White rot, causes butt and root rot.

Heilongjiang, Inner Mongolia, Jilin, Tibet, Xinjiang, and Yunnan.

***Phellinus tremulae*** **(Bondartsev) Bondartsev & P.N. Borisov**, polypore, Hymenochaetaceae, Hymenochaetales

Exclusively on *Populus dividiana* in native forests.

White rot, causes heartwood rot.

Northeast and northwest China.

***Pholiota squarrosa*** **(Vahl) P. Kumm**., agaric, Strophariaceae, Agaricales

Mostly on *Populus* and *Betula*, but also on several angiosperm tree species in native forests, plantations, and gardens.

White rot, causes heartwood rot.

Temperate China.

***Phylloporia bibulosa*** **(Lloyd) Ryvarden**, polypore, Hymenochaetaceae, Hymenochaetales

On angiosperm tree species in native forests.

White rot, causes butt and root rot.

Guangdong and Zhejiang.

*Phylloporia clausenae **L.W. Zhou**, polypore, Hymenochaetaceae, Hymenochaetales

Mostly on *Clausena* in native forests.

White rot, causes heartwood rot.

Hainan and tropical Yunnan.

***Phylloporia crataegi*** **L.W. Zhou & Y.C. Dai**, polypore, Hymenochaetaceae, Hymenochaetales

Exclusively on *Crataegus* in plantations and gardens.

White rot, causes butt and root rot.

Liaoning.

*Phylloporia cylindrispora **L.W. Zhou**, polypore, Hymenochaetaceae, Hymenochaetales

On angiosperm tree species in native forests.

White rot, causes heartwood rot.

Guangxi.

*Phylloporia cystidiolophora **F. Wu, G.J. Ren & Y.C. Dai**, polypore, Hymenochaetaceae, Hymenochaetales

On angiosperm tree species in native forests.

White rot, causes heartwood rot.

Chongqing and Yunnan.

*Phylloporia flacourtiae **L.W. Zhou**, polypore, Hymenochaetaceae, Hymenochaetales

On *Flacourtia* in native forests.

White rot, causes heartwood rot.

Guangxi.

***Phylloporia fontanesiae*** **L.W. Zhou & Y.C. Dai**, polypore, Hymenochaetaceae, Hymenochaetales

Exclusively on *Fontanesia* in native forests, plantations, and gardens.

White rot, causes butt and root rot.

Henan, Jiangsu, and Shandong.

***Phylloporia gutta*** **L.W. Zhou & Y.C. Dai**, polypore, Hymenochaetaceae, Hymenochaetales

Mostly on *Abelia* in native forests.

White rot, causes butt and root rot.

Beijing, Hebei, Inner Mongolia, and Sichuan.

***Phylloporia hainaniana*** **Y.C. Dai & B.K. Cui**, polypore, Hymenochaetaceae, Hymenochaetales

On angiosperm tree species in native forests.

White rot, causes heartwood rot.

Hainan.

*Phylloporia homocarnica **L.W. Zhou**, polypore, Hymenochaetaceae, Hymenochaetales

On angiosperm tree species in native forests.

White rot, causes heartwood rot.

Guangxi.

*Phylloporia lespedezae **G.J. Ren & F. Wu**, polypore, Hymenochaetaceae, Hymenochaetales

On *Lespedeza bicolour* in native forests.

White rot, causes root rot.

Shanxi.

*Phylloporia lonicerae **W.M. Qin, Xue W. Wang, T. Sawahata & L.W. Zhou**, polypore, Hymenochaetaceae, Hymenochaetales

On *Lonicera japonica* in plantations and gardens.

White rot, causes heartwood rot.

Shandong.

*Phylloporia manglietiae **Yuan Y. Chen & B.K. Cui**, polypore, Hymenochaetaceae, Hymenochaetales

On *Manglietiae hainanensis* in native forests.

White rot, causes heartwood rot.

Hainan.

*Phylloporia minutipora **L.W. Zhou**, polypore, Hymenochaetaceae, Hymenochaetales

On *Nephelium topengii* in native forests.

White rot, causes butt and root rot.

Hainan.

***Phylloporia nandinae*** **L.W. Zhou & Y.C. Dai**, polypore, Hymenochaetaceae, Hymenochaetales

Exclusively on *Nandina domestica* in plantations and gardens.

White rot, causes butt and root rot.

Fujian, Guizhou, Jiangxi, and Sichuan.

***Phylloporia oblongospora*** **Y.C. Dai & H.S. Yuan**, polypore, Hymenochaetaceae, Hymenochaetales

On angiosperm tree species in native forests.

White rot, causes butt and root rot.

Guangdong and Guangxi.

***Phylloporia oreophila*** **L.W. Zhou & Y.C. Dai**, polypore, Hymenochaetaceae, Hymenochaetales

Mostly on *Prinsepia* in native forests.

White rot, causes heartwood rot.

Tibet.

*Phylloporia osmanthi **L.W. Zhou**, polypore, Hymenochaetaceae, Hymenochaetales

On *Osmanthus* in native forests.

White rot, causes heartwood rot.

Guangxi.

*Phylloporia pendula **Yuan Y. Chen & B.K. Cui**, polypore, Hymenochaetaceae, Hymenochaetales

On angiosperm tree species in native forests.

White rot, causes heartwood rot.

Hainan.

*Phylloporia radiata **L.W. Zhou**, polypore, Hymenochaetaceae, Hymenochaetales

On lianas in native forests.

White rot, causes butt and root rot.

Guangdong.

*Phylloporia rattanicola **F. Wu, G.J. Ren & Y.C. Dai**, polypore, Hymenochaetaceae, Hymenochaetales

On rattan in native forests.

White rot, causes root rot.

Fujian.

*Phylloporia splendida **F. Wu, G.J. Ren & Y.C. Dai**, polypore, Hymenochaetaceae, Hymenochaetales

On angiosperm tree species in native forests.

White rot, causes heartwood rot.

Yunnan and Zhejiang.

*Phylloporia subpulla **F. Wu, G.J. Ren & Y.C. Dai**, polypore, Hymenochaetaceae, Hymenochaetales

On angiosperm tree species in native forests.

White rot, causes heartwood rot.

Hainan.

***Phylloporia weberiana*** **(Bres. & Henn. ex Sacc.) Ryvarden**, polypore, Hymenochaetaceae, Hymenochaetales

On angiosperm tree species in native forests.

White rot, causes heartwood rot.

Hainan.

***Picipes fraxinicola*** **(L.W. Zhou & Y.C. Dai) J.L. Zhou & B.K. Cui**, polypore, Polyporaceae, Polyporales

On *Quercus*, especially on *Q*. *mongolica* in old-growth native forests.

White rot, causes heartwood rot.

Jilin.

***Piptoporellus soloniensis*** **(Dubois) B.K. Cui, M.L. Han & Y.C. Dai**, polypore, Piptoporellaceae, Polyporales

Mostly on *Castanea* and *Quercus* in both native forests and plantations.

Brown rot, causes heartwood rot.

Fujian, Hubei, Hunan, Jilin, Liaoning, Shandong, Sichuan, and Yunnan.

***Pleurotus ostreatus*** **(Jacq.) P. Kumm**., agaric, Pleurotaceae, Agaricales

Mostly on *Populus*, *Salix*, or *Betula* in native forests, plantations, and gardens.

White rot, causes heartwood rot.

Temperate China.

***Polyporus squamosus*** **(Huds.) Fr**., polypore, Polyporaceae, Polyporales

On many angiosperm tree species, especially on *Fraxinus*, *Quercus*, and *Ulmus* in native forests, plantations, and gardens.

White rot, causes and heartwood rot.

Beijing and temperate China.

***Polyporus subvarius*** **C.J. Yu & Y.C. Dai**, polypore, Polyporaceae, Polyporales

Mostly on *Salix* in plantations and gardens.

White rot, causes heartwood rot.

Tibet.

*Porodaedalea alpicola **S.J. Dai, F. Wu & Y.C. Dai**, polypore, Hymenochaetaceae, Hymenochaetales

Exclusively on *Abies* in native forests.

White rot, causes heartwood rot.

Sichuan, Tibet, and Yunnan.

***Porodaedalea himalayensis*** **(Y.C. Dai) Y.C. Dai**, polypore, Hymenochaetaceae, Hymenochaetales

Mostly on *Picea* in native forests.

White rot, causes heartwood rot.

Gansu, northern Yunnan, Sichuan, and Tibet.

***Porodaedalea laricis*** **(Jacz. ex Pilát) Niemelä**, polypore, Hymenochaetaceae, Hymenochaetales

Mostly on *Larix* in native forests.

White rot and heartwood rot.

Hebei, Heilongjiang, Inner Mongolia, Jilin, and Xinjiang.

*Porodaedalea microsperma **S.J. Dai & Y.C. Dai**, polypore, Hymenochaetaceae, Hymenochaetales

On *Picea* and *Larix* in native forests.

White rot, causes heartwood rot.

Heilongjiang, Inner Mongolia, and Jilin.

*Porodaedalea mongolica **Y.D. Wu & Y. Yuan**, polypore, Hymenochaetaceae, Hymenochaetales

Exclusively on *Larix* in native forests.

White rot, causes heartwood rot.

Heilongjiang and Inner Mongolia.

*Porodaedalea qilianensis **Y.C. Dai & F. Wu**, polypore, Hymenochaetaceae, Hymenochaetales

Exclusively on *Picea crassifolia* in native forests.

White rot, causes heartwood rot.

Gansu and Qinghai.

*Porodaedalea schrenkianae **Y.C. Dai & F. Wu**, polypore, Hymenochaetaceae, Hymenochaetales

Exclusively on *Picea schrenkiana* in native forests.

White rot, causes heartwood rot.

Xinjiang.

***Porodaedalea yamanoi*** **(Imazeki) Y.C. Dai**, polypore, Hymenochaetaceae, Hymenochaetales

Mostly on *Picea* in native forests.

White rot, causes heartwood rot.

Heilongjiang and Jilin.

*Porodaedalea yunnanensis **S.J. Dai, F. Wu & Y.C. Dai**, polypore, Hymenochaetaceae, Hymenochaetales

Exclusively on *Pinus yunnanensis* in native forests.

White rot, causes heartwood rot.

Yunnan.

***Pseudoinonotus tibeticus*** **(Y.C. Dai & M. Zang) Y.C. Dai, B.K. Cui & Decock**, polypore, Hymenochaetaceae, Hymenochaetales

On *Abies* in old growth native forests.

White rot, causes heartwood rot.

Tibet and Yunnan.

***Pseudospongipellis litschaueri*** **(Lohwag) Y.C. Dai & Chao G. Wang**, polypore, Cerrenaceae, Polyporales

Mostly on *Quercus* in native forests, plantations, and gardens.

White rot, causes heartwood rot.

Heilongjiang, Hubei, Jilin, Liaoning, and Shanxi.

***Pyrofome*****s *castanopsidis* B.K. Cui & Y.C. Dai**, polypore, Polyporaceae, Polyporales

Exclusively on *Castanopsis* in native forests and gardens.

White rot, causes heartwood rot.

Guangdong.

***Pyrofomes demidoffii*** **(Lév.) Kotl. & Pouzar**, polypore, Polyporaceae, Polyporales

Exclusively on *Juniperus formosana* in plantations and gardens.

White rot, causes heartwood rot.

Sichuan.

***Pyrrhoderma sublamaensis*** **(Lloyd) Y.C. Dai & F. Wu**, polypore, Hymenochaetaceae, Hymenochaetales

Mainly on *Hevea brasilensis*, but also on many other angiosperm tree species in plantations.

White rot, causes root rot.

Guangdong, Guangxi, Hainan, and Yunnan.

***Rigidonotus pruinosus*** **(Bondartsev) Y.C. Dai, F. Wu, L.W. Zhou, Vlasák & B.K. Cui**, polypore, Hymenochaetaceae, Hymenochaetales

Exclusively on *Salix* in plantations and gardens.

White rot, causes heartwood rot.

Liaoning.

***Rigidoporus lineatus*** **(Pers.) Ryvarden**, polypore, Meripilaceae, Polyporales

On many angiosperm tree species, especially on *Robinia* and *Paulownia* in plantations and gardens.

White rot, causes butt and root rot.

Fujian, Guangxi, Guizhou, Henan, Hubei, Hunan, Jiangsu, and Yunnan.

***Rigidoporus microporus*** **(Sw.) Overeem**, polypore, Meripilaceae, Polyporales

On many angiosperm tree species in native forests, plantations, and gardens.

White rot, causes butt and root rot.

Hainan and Yunnan.

***Rigidoporus populinus*** **(Schumach.) Pouzar**, polypore, Meripilaceae, Polyporales

Mostly on *Acer* and *Populus*, occasionally on other angiosperm tree species in native forests, plantations, and gardens.

White rot, causes heartwood rot.

Boreal and temperate China.

***Rigidoporus subpopulinus*** **(B.K. Cui & Y.C. Dai) F. Wu, Jia J. Chen & Y.C. Dai**, polypore, Meripilaceae, Polyporales

Exclusively on *Picea crassifolia* in old-growth native forests.

White rot, causes heartwood rot.

Gansu and Qinghai.

*Sanghuangporus alpinus **(Y.C. Dai & X.M. Tian) L.W. Zhou & Y.C. Dai**, polypore, Hymenochaetaceae, Hymenochaetales

Exclusively on *Lonicera rupicola* in native forests.

White rot, causes heartwood rot.

Northern Yunnan, Tibet, and western Sichuan.

***Sanghuangporus baumii*** **(Pilát) L.W. Zhou & Y.C. Dai**, polypore, Hymenochaetaceae, Hymenochaetales

Almost exclusively on *Syringa amurensis* in native forests, plantations, and gardens.

White rot, causes heartwood rot.

Beijing, Hebei, Heilongjiang, Inner Mongolia, Jilin, and Liaoning.

*Sanghuangporus lagerstroemiae **Y.C. Dai & F. Wu**, polypore, Hymenochaetaceae, Hymenochaetales

On several angiosperm tree species, e.g. *Lagerstroemia*, *Cryptocarya*, and *Quercus* in native forests and gardens.

White rot, causes heartwood rot.

Beijing and Sichuan.

***Sanghuangporus lonicericola*** **(Parmasto) L.W. Zhou & Y.C. Dai**, polypore, Hymenochaetaceae, Hymenochaetales

Exclusively on *Lonicera syringantha* and *L*. *maackii* in native forests, very rarely in plantations.

White rot, causes heartwood rot.

Heilongjiang, Jilin, Liaoning, and Tibet.

*Sanghuangporus mongolicus **T. Bau**, polypore, Hymenochaetaceae, Hymenochaetales

Exclusively on *Hemiptelea davidii* in plantations.

White rot, causes heartwood rot.

Inner Mongolia.

*Sanghuangporus quercicola **Lin Zhu & B.K. Cui**, polypore, Hymenochaetaceae, Hymenochaetales

Mostly on *Quercus*, occasionally on *Diospyros* in native forests and plantations.

White rot, causes heartwood rot.

Chongqing, Guizhou, Henan, and Tibet.

***Sanghuangporus sanghuang*** **(Sheng H. Wu, T. Hatt. & Y.C. Dai) Sheng H. Wu, L.W. Zhou & Y.C. Dai**, polypore, Hymenochaetaceae, Hymenochaetales

Exclusively on *Morus* in native forests, plantations, and gardens.

White rot, causes heartwood rot.

Hubei, Jilin, Shaanxi, Shanxi, Sichuan, Tibet, and Zhejiang.

*Sanghuangporus subbaumii **Shan Shen, Y.C. Dai & L.W. Zhou**, polypore, Hymenochaetaceae, Hymenochaetales

Mostly on *Prunus* in native forests and plantations.

White rot, causes heartwood rot.

Beijing and Shanxi.

***Sanghuangporus vaninii*** **(Ljub.) L.W. Zhou & Y.C. Dai**, polypore, Hymenochaetaceae, Hymenochaetales

Exclusively on *Populus* in native forests.

White rot, causes heartwood rot.

Beijing, Heilongjiang, Jilin, and Shanxi.

*Sanghuangporus viticicola **Sheng H. Wu**, polypore, Hymenochaetaceae, Hymenochaetales

On *Vitex* in native forests.

White rot, causes heartwood rot.

Taiwan Province of China.

***Sanghuangporus weigela*****e (T. Hatt. & Sheng H. Wu) Sheng H. Wu, L.W. Zhou & Y.C. Dai**, polypore, Hymenochaetaceae, Hymenochaetales

Mostly on *Weigela* in native forests, plantations, and gardens.

White rot, causes heartwood rot.

Anhui, Chongqing, Guizhou, Hubei, Hunan, Inner Mongolia, Jiangxi, and Zhejiang.

*Sanghuangporus zonatus **(Y.C. Dai & X.M. Tian) L.W. Zhou & Y.C. Dai**, polypore, Hymenochaetaceae, Hymenochaetales

On angiosperm tree species in native forests.

White rot, causes heartwood rot.

Hainan.

***Sanguinoderma elmerianum*** **(Murrill) Y.F. Sun & B.K. Cui**, polypore, Ganodermataceae, Polyporales

Mostly on *Acacia*, especially on *A*. *richii*, sometimes on other angiosperm tree species in plantations and gardens.

White rot, causes butt rot.

Fujian, Guangdong, Guangxi, and Hainan.

***Schizophyllum commune*** **Fr**., agaric, Schizophyllaceae, Agaricales

On many angiosperm tree species, especially on *Prunus* in plantations and gardens.

White rot, causes sapwood rot.

Temperate China.

***Sparassis latifolia*** **Y.C. Dai & Zheng Wang**, corticioid, Sparassidaceae, Polyporales

On *Larix* in old growth native forests.

Brown rot, causes root rot.

Jilin, Tibet, and Yunnan.

*Sparassis subalpina **Q. Zhao, Zhu L. Yang & Y.C. Dai**, corticioid, Sparassidaceae, Polyporales

On *Picea* in old growth native forests.

Brown rot, causes root rot.

Northern Yunnan.

*Spongipellis quercicola **Y.C. Dai & Chao G. Wang**, polypore, Meruliaceae, Polyporales

On *Quercus mongolica* in old growth native forests.

White rot, causes heartwood rot.

Jilin.

***Spongipellis spumea*** **(Sowerby) Pat**., polypore, Meruliaceae, Polyporales

On many angiosperm tree species, especially *Populus* in native forests, plantations, and gardens.

White rot, causes heartwood rot.

Temperate China.

***Stereum rugosum*** **Pers**., corticioid, Stereaceae, Russulales

On many angiosperm tree species in native forests, plantations, and gardens.

White rot, causes sapwood rot.

Boreal and temperate China.

***Stereum sanguinolentum*** **(Alb. & Schwein.) Fr**., corticioid, Stereaceae, Russulales

On many gymnosperm tree species, especially on *Picea*, *Pinus,* and *Larix* in native forests.

White rot, causes sapwood rot.

Boreal and temperate China.

***Trachydermella tsunodae*** **(Yasuda ex Lloyd) B.K. Cui & Y.F. Sun**, polypore, Ganodermataceae, Polyporales

Mainly on *Litsea cubeba* in native forests.

White rot, causes butt and root rot.

Guizhou and Yunnan.

***Trametes meyenii*** **(Klotzsch) Lloyd**, polypore, Polyporaceae, Polyporales

On many angiosperm trees in native forests.

White rot, causes butt rot.

Subtropical China.

***Trametes suaveolens*** **(L.) Fr**., polypore, Polyporaceae, Polyporales

On many angiosperm tree species, especially on *Salix* and *Populus* in native forests, plantations, and gardens.

White rot, causes heartwood rot.

Boreal and temperate China.

***Trichaptum perenne*** **Y.C. Dai & H.S. Yuan**, polypore, Hymenochaetaceae, Hymenochaetales

Mostly on *Lithocarpus* in old-growth native forests.

White rot, causes heartwood rot.

Central Yunnan.

*Tropicoporus tenuis **Y.C. Dai & F. Wu**, polypore, Hymenochaetaceae, Hymenochaetales

On angiosperm tree species in native forests.

White rot, causes heartwood rot.

Guangdong.

***Tyromyces sibiricus*** **Penzina & Ryvarden**, polypore, Incrustoporiaceae, Polyporales

Mostly on *Populus* and *Acer* in old growth native forests.

White rot, causes heartwood rot.

Heilongjiang and Jilin.

***Vanderbylia fraxinea*** **(Bull.) D.A. Reid**, polypore, Polyporaceae, Polyporales

On many angiosperm tree species in native forests, plantations, and gardens.

White rot, causes butt and root rot.

Temperate to subtropical China.

***Vanderbylia robiniophila*** **(Murrill) B.K. Cui & Y.C. Dai**, polypore, Polyporaceae, Polyporales

Mostly on *Robinia pseudoacacia* in plantations and gardens.

White rot, causes butt and root rot.

Beijing, Jiangsu, Liaoning, Shandong, and Shanxi.

## 4. Discussion

Pathogenic wood-rotting basidiomycetes have important implications in the forestry industry, for instance, some species of *Heterobasidion* Bref. cause extensive root rot on coniferous trees in the Northern Hemisphere (Woodward et al. 1998). These pathogenic species are of interest not only to forest pathologists but also to taxonomists (Sinclair et al. 1987; Schulze and Bahnweg 1998; Fischer and Binder 2004; Sinclair and Lyon 2005; Fischer et al. 2005; Linzer et al. 2008; Otrosina and Garbelotto 2010; Rajchenberg and Robledo 2013; Yuan et al. 2021).

Among 205 pathogenic wood-rotting species, some of them cause typical sapwood or heartwood or root and butt rot according to our investigations, but their pathogenicity is not proved by the rule of Koch’s postulate, and further studies are needed in the future to verify the pathogenicity. About 1,600 wood-rotting basidiomycetes are found in China, and 205 species were found on living trees, and they are considered as pathogenic species. So, the pathogenic species accounts for 12.8% of all the Chinese wood-rotting species. Similar cases were demonstrated in Europe and North America, 65 and 68 poroid wood-rotting basidiomycetes were considered as forest pathogens in Europe and North America (Gilbertson and Ryvarden 1986–1987; Ryvarden and Melo 2014), and 547 and 432 wood-rotting polypores were reported in Europe and North America, respectively (Wu et al. 2022), the pathogenic species accounts for 11.8% and 15.7% of all the total polypores in Europe and in North America.

Previously, 152 pathogenic wood-rotting basidiomycetes were recorded in China (Dai 2012), including the following 17 species (Dai et al. 2007; Dai 2012), *Bondarzewia berkeleyi* (Fr.) Bondartsev & Singer, *B. montana* (Quél.) Singer, *Dichomitus campestris* (Quél.) Domański & Orlicz, *Flammulina velutipes* (Curtis) Singer, *Fomitiporia dryadea* (Pers.) Y.C. Dai, *Fomitiporia hippophaëicola* (H. Jahn) Fiasson & Niemelä, *Fomitiporia robusta* (P. Karst.) Fiasson & Niemelä, *Fomitopsis pinicola* (Sw.) P. Karst., *Ganoderma pseudoferreum* (Wakef.) Over. & Steinm., *Heterobasidion parviporum* Niemelä & Korhonen, *Inonotus andersonii* (Wll. & Everh.) Černý, *Onnia triquetra* (Lenz) Imazeki, *Phellinidium weirii* (Murrill) Y.C. Dai, *Phellinus pini* (Fr.) A. Ames, *P. pseudoigniarius* Y.C. Dai & Fan Yang, *P. tuberculosus* (Baumg.) Niemelä, *Phylloporia ribis* (Schumach.) Ryvarden. However, recent studies confirm that these species do not exist in China (Chen et al. 2016; Zhou et al. 2016; Ji et al. 2017; Wu et al. 2019, 2022; Liu et al. 2021, 2023; Yuan et al. 2021, 2022). In addition, 72 species are found on living trees and cause sapwood or heartwood or butt and root rot, but they were not listed in the previous studies, and are new forest pathogens in China. To date, the number of pathogenic wood-rotting species found in China has reached 205.

**Figure 5.**
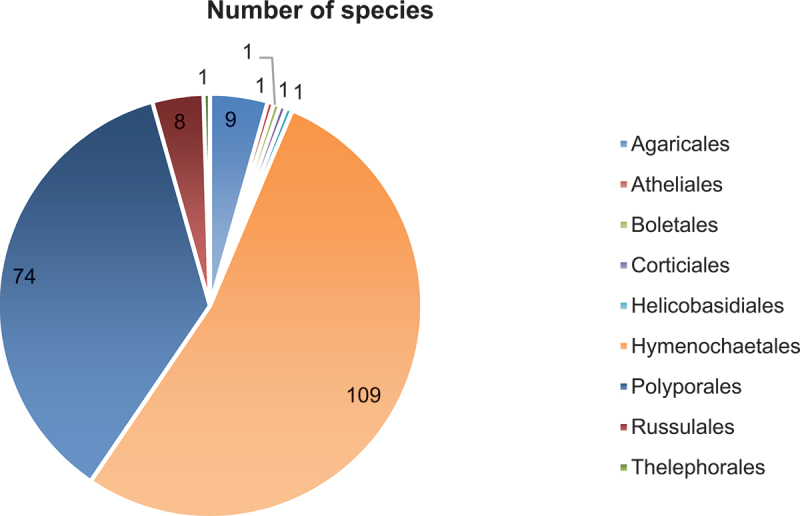
Pathogenic wood-rotting species in orders and number of species.

**Figure 6.**
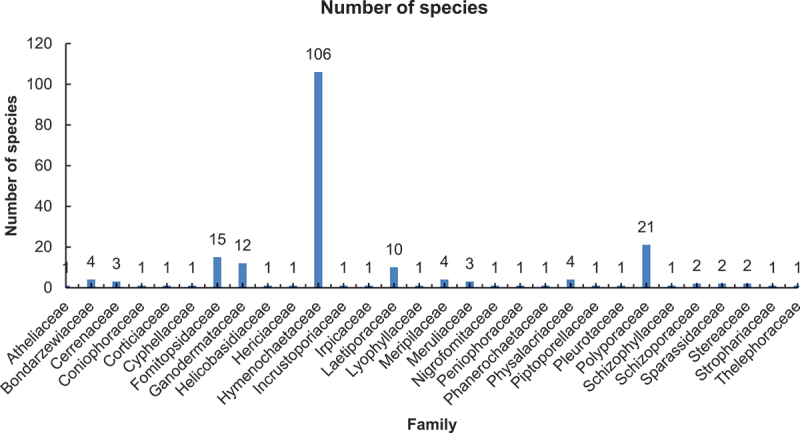
Pathogenic wood-rotting species in families and number of species.

The 205 pathogenic wood-rotting species belong to 9 orders, 30 families, and 74 genera (Figures 5–6). Hymenochaetales with 109 species, Hymenochaetaceae with 106 species, and *Phylloporia* with 24 species are, respectively, the richest order, family, and genus for the pathogenic wood-rotting basidiomycetes. The important families for these pathogens are Hymenochaetaceae, Polyporaceae, Fomitopsidaceae, Ganodermataceae, and Laetiporaceae, with 106, 21, 15, 12, and 10 species, respectively. A total of 164 species belong to these five families, and these species account for 80% of all Chinese pathogenic wood-rotting basidiomycetes.

Among the 205 species, 183 are polypores, 9 are corticioid fungi, 8 are agarics, and 5 are hydnoid basidiomycetes. So, polypores are the most important pathogenic wood-rotting basidiomycetes. In addition, 177 species cause white rot, and 28 cause brown rot. The higher diversity of white rot species is because they are distributed in almost all Chinese forests and infect both angiosperm trees and gymnosperm trees, while brown rot species occur primarily on gymnosperms and are found mostly in temperate and boreal forests.

Among the 205 species, 95 were found in boreal to temperate and 110 were found in subtropical to tropical forests of China, because the tree species diversity in southern China is greater than in northern China. One hundred and fifty-seven species occur on angiosperm trees and 44 species occur on gymnosperm trees, accounting for 76.5% and 21.5%, respectively, of the total pathogenic wood-rotting species. In addition, four species grow on both angiosperms and gymnosperms, accounting for 2% of the total pathogenic wood-rotting species. This shows that most pathogenic wood-rotting basidiomycetes have host-preference at least at the angiosperm or gymnosperm level.
